# In this issue: Volume 114, Issue 6, June 2023

**DOI:** 10.1111/cas.15854

**Published:** 2023-05-22

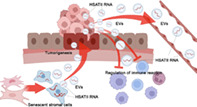

## Recent advances in extracellular vesicles in gastrointestinal cancer and lymphoma

Extracellular vesicles (EVs) have long been known to exist, but only as “trash bags” or cellular wastes with no essential biological function. However, since the discovery of microRNAs (miRNAs) in EVs, they have piqued the interest of cancer biologists. Tumor cells secrete a large number of EVs, which can be used in ‘liquid biopsy’ procedures for cancer detection. Recently EVs have also been found to contain non‐binding RNAs, like long non‐coding RNAs (lncRNAs), circular RNAs (circRNAs), and repeat RNAs, which can be easily amplified and used for detecting cancer types.

EVs also deliver bioactive molecules like lipids and proteins to target cells, which may promote metastases i.e., the spread of cancer. These molecules may be used in combination with other markers to create sensitive and specific EV‐based diagnostics. It is, therefore, speculated that EVs carry significant diagnostic value for various cancers.

In this review, Otsuka and colleague describe how EVs participate in gastrointestinal cancer pathogenesis and how their bioactive cargoes exhibit excellent diagnostic utility. The authors also discuss how human satellite II (HSATII) RNA, a ncRNA found in EVs, suppresses anticancer immune surveillance and can be used as a biomarker to detect pancreatic and colon cancer. They go on to explain how lipids, phosphatidylserine, and phospholipids found in EVs contribute to cell‐to‐cell communication and influence the tumor microenvironment.

Phospholipids are broken down to fatty acids and other substances by enzymes called phospholipases. The hydrolysis of phospholipases can increase small EV (sEV) uptake by target cells and also transduce biological signals to immune cells, which do not take up sEVs. This has been proposed as a new sEV signaling mechanism. A secreted phospholipase A2 (sPLA2) and its isoform sPLA2‐IIA have been found to protect against gastrointestinal cancer by its tumor‐suppressing action. However, the same enzyme is also involved in promoting prostate cancer. Another sPLA2, called sPLA2‐X, breaks down tumor‐derived sEVs in the human lymphomas, thereby promoting tumor formation.

The above facts clearly suggest that EVs may promote or inhibit tumor progression in different cancer types. Moreover, some EVs from cancer cells may interact directly with therapeutic agents, reducing their efficacy. As a result, their biological significance, utility as cancer markers, and potential as treatment targets must be carefully evaluated and considered.

To summarize, EVs can serve as next‐generation cancer biomarkers and contribute in advancing cancer diagnostics and therapy. In addition, controlling cancer‐derived EVs may lead to the development of novel therapeutics. However, this is a new field of research which warrants meticulous investigation of EV function, particularly aimed at cancer suppression and inhibition.

https://onlinelibrary.wiley.com/doi/10.1111/cas.15771

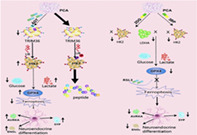

### Trigred motif 36 regulates neuroendocrine differentiation of prostate cancer via HK2 ubiquitination and GPx4 deficiency

Neuroendocrine prostate cancer (NEPC) is a severe form of prostate cancer, often attributed to the prolonged use of androgen deprivation therapy (ADT). Ironically, ADT is the most frequently used treatment for aggressively spreading prostate cancer. Incidence of NEPC is predicted to increase rapidly due to the growing use of new ADT. This makes it important to understand the development of NEPC to develop effective therapies against it.

Previous studies have identified Trigred motif 36 (TRIM36) as a gene that responds to androgens (such as testosterone) and plays a tumor suppressive role in prostate cancer. Now, in a new study, Zhao et al. identified the role of TRIM36 in the development of NEPC using mass spectrometry‐based quantitative protein studies. The authors also shed light on the association of TRIM36 in the neuroendocrine differentiation of prostate cancer cells involving glycolysis, the cellular energy metabolic pathway.

Through extensive proteomic analysis, Zhao et al. observed that TRIM36 expression is significantly reduced in NEPC as compared to other forms of resistant prostate cancer. Further, they found that increased levels of TRIM36 hampered the glycolysis pathway by binding to and ubiquitinated the glycolytic enzyme, hexokinase 2 (HK2). Decreased levels of HK2 led to a decrease in the expression of an enzyme responsible for reducing the oxidative stress within the cells, called glutathione peroxidase 4, thus resulting in ferroptosis or iron‐dependent cell death.

The findings of this study suggest that glycolysis suppression and ferroptosis activation together regulate the neuroendocrine differentiation of prostate cancer, which are affected by the presence or absence of TRIM36. Thus, TRIM36 may be useful in preventing neuroendocrine differentiation, and can act as a potential therapeutic marker in the development of new treatments against NEPC.

https://onlinelibrary.wiley.com/doi/10.1111/cas.15763

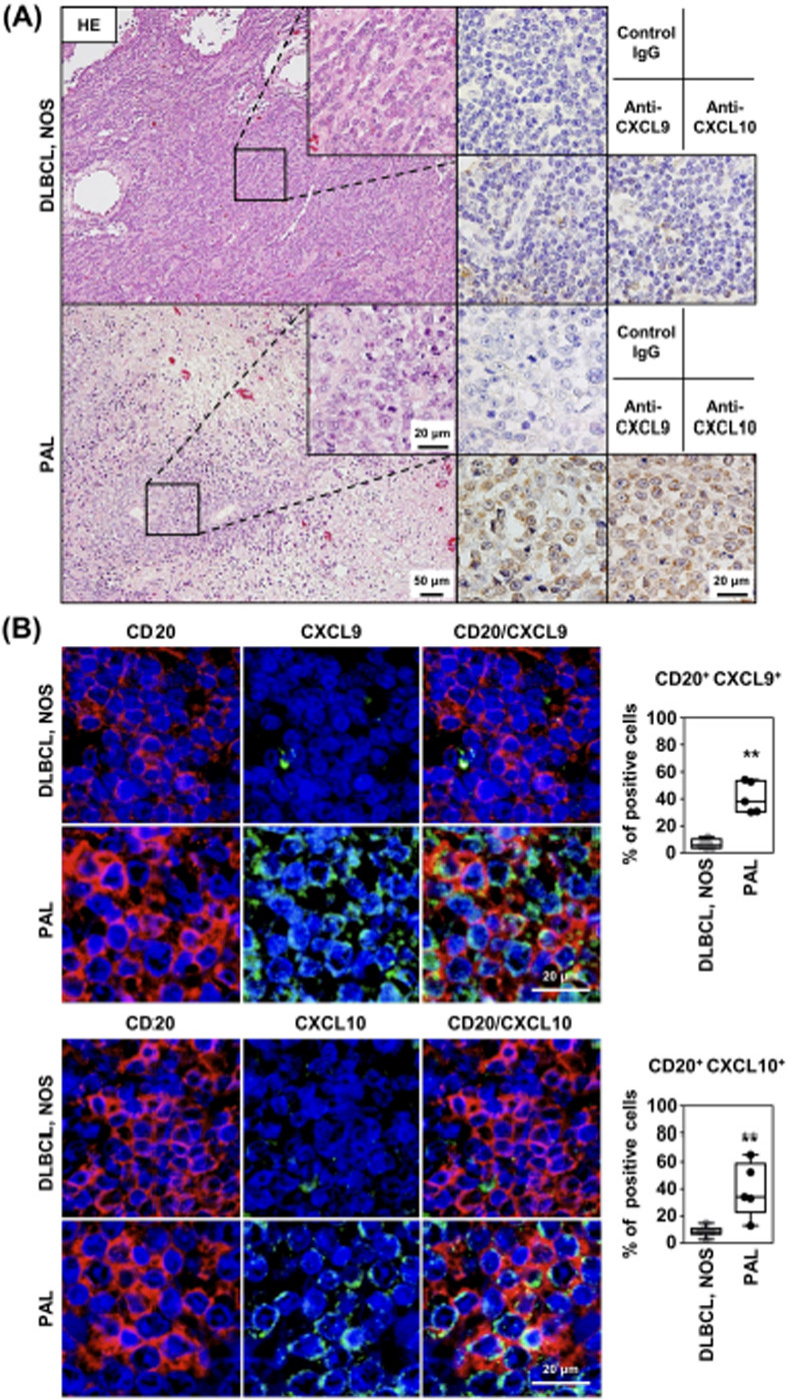

### EBV‐positive pyothorax‐associated lymphoma expresses CXCL9 and CXCL10 chemokines that attract cytotoxic lymphocytes via CXCR3

The Epstein Barr virus (EBV)‐positive diffuse large B‐cell lymphoma associated with chronic inflammation (DLBCL‐CI) is a type of cancer that develops after a period of chronic inflammation. DLBCL‐CI can be studied using its prototype, called “pyothorax‐associated lymphoma” (PAL). However, it is unknown how PAL develops from chronic inflammation.

Chemokines are molecules that respond to inflammatory signals. They activate and recruit immune cells that then mediate defense responses. The C–X–C motif chemokines – CXCL9, CXCL10, and CXCL11 – play important roles in cytotoxic lymphocyte trafficking and activate the chemokine receptor 3 (CXCR3) found on immune cells. While the CXCR3 ligand exerts antitumor effects and destroys cellular tissue, also referred to as “tissue necrosis,” it is speculated that the CXC chemokines may be involved in the pathogenesis of PAL.

In this article, Higuchi et al. investigated if PAL cells can produce CXCR3 ligands that attract the CXCR3‐expressing effector cells. They examined the expression of chemokines in six EBV‐positive PAL cell lines. Unlike EBV‐negative DLBCL cell lines, the cell lines were found to express and secrete CXCL9 and CXCL10. The supernatant of these cell lines could attract CXCR3‐expressing lymphocytes from the blood, such as CD4^+^ T cells, CD8^+^ T cells, and CD56^+^ killer cells. This showed that the antitumor chemokines were functional in the supernatant.

These findings were then validated in vivo using mice injected with PAL cells. As expected, PAL cells attracted the CXCR3‐positive cytotoxic lymphocytes that expressed interferon‐γ (a cytotoxic immune cell marker). Finally, the authors obtained tumor biopsies from patients with PAL and found strongly expressed CXCL9 and CXCL10. Additionally, abundant CXCR3‐expressing lymphocytes were detected in the tissue samples, implying the cytotoxic effects of these chemokines.

Overall, this study shows that PAL cells can produce CXCL9 and CXCL10, which attract CXCR3‐expressing cytotoxic lymphocytes to the inflammatory and tumor microenvironments to elicit tissue necrosis. The authors suggest that the pathogenesis of tissue necrosis in DLBCL could be linked to the CXCR3 axis, which may potentially be involved in the activation of antitumor immunity.

Given that DLBCL is one of the most common and aggressive cancers, these findings could help facilitate new therapeutic strategies for activating antitumor immunity in patients with treatment‐resistant DLBCL.

https://onlinelibrary.wiley.com/doi/10.1111/cas.15782

